# A BLUS1 kinase signal and a decrease in intercellular CO_2_ concentration are necessary for stomatal opening in response to blue light

**DOI:** 10.1093/plcell/koab067

**Published:** 2021-03-01

**Authors:** Sakurako Hosotani, Shota Yamauchi, Haruki Kobayashi, Saashia Fuji, Shigekazu Koya, Ken-ichiro Shimazaki, Atsushi Takemiya

**Affiliations:** 1 Department of Biology, Graduate School of Sciences and Technology for Innovation, Yamaguchi University, Yamaguchi 753-8512, Japan; 2 Department of Biology, Kyushu University, Fukuoka 819-0395, Japan

## Abstract

Light-induced stomatal opening stimulates CO_2_ uptake and transpiration in plants. Weak blue light under strong red light effectively induces stomatal opening. Blue light-dependent stomatal opening initiates light perception by phototropins, and the signal is transmitted to a plasma membrane H^+^-ATPase in guard cells via BLUE LIGHT SIGNALING 1 (BLUS1) kinase. However, it is unclear how BLUS1 transmits the signal to H^+^-ATPase. Here, we characterized BLUS1 signaling in *Arabidopsis thaliana*, and showed that the BLUS1 C-terminus acts as an auto-inhibitory domain and that phototropin-mediated Ser-348 phosphorylation within the domain removes auto-inhibition. C-Terminal truncation and phospho-mimic Ser-348 mutation caused H^+^-ATPase activation in the dark, but did not elicit stomatal opening. Unexpectedly, the plants exhibited stomatal opening under strong red light and stomatal closure under weak blue light. A decrease in intercellular CO_2_ concentration via red light-driven photosynthesis together with H^+^-ATPase activation caused stomatal opening. Furthermore, phototropins caused H^+^-ATPase dephosphorylation in guard cells expressing constitutive signaling variants of BLUS1 in response to blue light, possibly for fine-tuning stomatal opening. Overall, our findings provide mechanistic insights into the blue light regulation of stomatal opening.

## Teaser

BLUE LIGHT SIGNALING 1-mediated H^+^-ATPase activation and a decrease in intercellular CO_2_ concentration via red light-driven photosynthesis are required for blue light-dependent stomatal opening.

## Introduction

Stomata are microscopic pores formed by pairs of specialized guard cells in the leaves of terrestrial plants. Strict regulation of stomatal opening is essential for terrestrial plants to uptake carbon dioxide (CO_2_) for photosynthesis, while balancing water loss via transpiration (Hetherington and Woodward, 2003; Roelfsema and Hedrich, 2005; Shimazaki et al., 2007; Kim et al., 2010; Lawson and Blatt, 2014; Assmann and Jegla, 2016). Plants have evolved at least two distinct mechanisms involved in guard cell signaling that ensure optimum stomatal opening under changing light environments, namely, blue light- and red light-induced responses (Shimazaki et al., 2007; Matthews et al., 2020). The blue light-dependent response is induced by a low fluence rate of blue light, owing to photoreceptor-coupled activation of proton pumps localized in the plasma membrane and the subsequent ion uptake by guard cells (Shimazaki et al., 2007; Inoue and Kinoshita, 2017). In contrast, the red light-induced response requires a continuous high fluence rate of red light and is inhibited by 3-(3,4-dichlorophenyl)-1, 1-dimethylurea (DCMU), an inhibitor of electron transfer in photosystem II; this indicates that this response relies on photosynthesis (Kuiper, 1964; Sharkey and Raschke, 1981). Furthermore, the stomatal response under both weak blue and strong red lights is greater than the sum of responses induced by the same intensity of monochromatic blue and red lights, respectively (Shimazaki et al., 2007; Matthews et al., 2020). In addition, the magnitude of stomatal opening under blue light depends on the intensity of background red light, and frequently, no response is observed when plant leaves are illuminated with only weak blue light (Karlsson, 1986; Assmann, 1988). This synergistic effect of blue and red light on stomatal opening can be attributed to the interaction between phototropin-mediated response and photosynthesis of both guard cell and mesophyll chloroplasts. It has been suggested that the effect involves the provisioning of ATP from guard cell chloroplasts and reduction in intercellular CO_2_ concentration (*Ci*) by mesophyll chloroplasts (Karlsson, 1986; Assmann, 1988; Lascève et al., 1993; Shimazaki et al., 2007; Suetsugu et al., 2014). However, it is not clear how blue and red lights synergistically drive stomatal opening.

**Figure:**
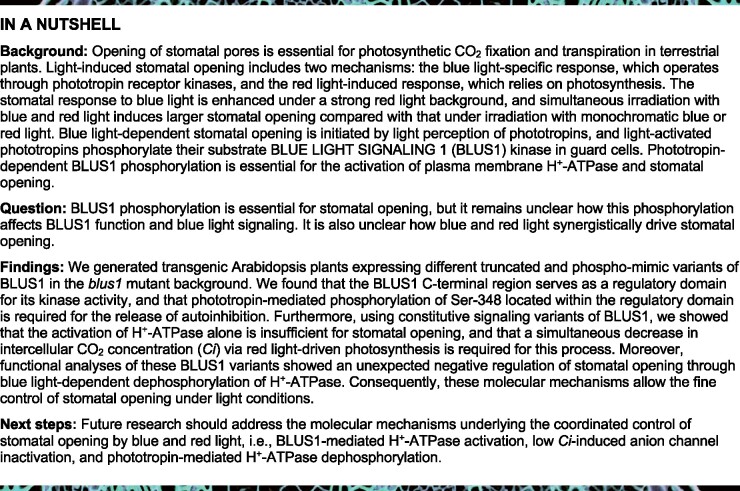


Blue light-dependent stomatal opening is initiated by light perception of photoreceptor kinase phototropins, phot1 and phot2 (Kinoshita et al., 2001). Phototropins contain two light-sensing Light, Oxygen, or Voltage (LOV) domains in the N-terminus and a Ser/Thr kinase domain in the C-terminus (Christie, 2007). Upon blue light perception, the chromophore flavin mononucleotide covalently binds to a conserved Cys residue within the LOV domain, leading to conformational changes that abate the repression of kinase activity by LOV2 (Christie, 2007). The light-activated phototropins autophosphorylate the kinase activation loop and phosphorylate their substrate BLUE LIGHT SIGNALING 1 (BLUS1) (Inoue et al., 2008; Takemiya et al., 2013a). The signal from BLUS1 induces the phosphorylation of the penultimate Thr residue of plasma membrane H^+^-ATPase with the subsequent binding of 14-3-3 proteins, and this alleviates the C-terminal auto-inhibitory effect and thus leads to H^+^-ATPase activation (Kinoshita and Shimazaki, 1999; Kinoshita and Shimazaki, 2002; Yamauchi et al., 2016). A recent study identified another phototropin kinase substrate CONVERGENCE OF BLUE LIGHT AND CO_2_ 1 (CBC1), a Raf-like MAP kinase kinase kinase (MAPKKK) (Hiyama et al., 2017). CBC1, along with its paralog, CBC2, inhibits S-type anion channels in response to blue light (Marten et al., 2007; Hiyama et al., 2017). Consequently, both activation of H^+^-ATPase and inactivation of anion channels cause plasma membrane hyperpolarization, facilitating the uptake of K^+^ via voltage-gated inward rectifying K^+^ channel and stomatal opening (Jezek and Blatt, 2017). Two signaling components, the Raf-like MAPKKK BLUE LIGHT-DEPENDENT H^+^-ATPASE PHOSPHORYLATION (BHP) and type 1 protein phosphatase (PP1), have been shown to act downstream of BLUS1 and upstream of H^+^-ATPase (Takemiya et al., 2006; Takemiya et al., 2013b; Hayashi et al., 2017). However, it is not clear how BLUS1 transfers the blue light signal for H^+^-ATPase activation.

The protein kinase BLUS1 was identified through forward genetic screens for the loss of blue light-dependent stomatal opening by infrared thermography (Takemiya et al., 2013a). It belongs to the germinal center kinase (GCK)-VI subfamily of Sterile 20 (Ste20)-related protein kinases and is highly conserved in angiosperms (Takemiya et al., 2013a; Sussmilch et al., 2019; Harris et al., 2020). BLUS1 comprises an N-terminal Ser/Thr kinase domain and a C-terminal uncharacterized domain. Phototropins interact with the kinase domain of BLUS1 irrespective of blue light and directly phosphorylate Ser-348 within the C-terminal domain of BLUS1 in response to blue light (Takemiya et al., 2013a, 2016). Both phot1 and phot2 phosphorylate BLUS1 at Ser-348, but phot1 phosphorylates BLUS1 more efficiently than the phot2 in guard cells (Takemiya and Shimazaki, 2016). A mutational analysis indicates that BLUS1 phosphorylation is essential for stomatal opening (Takemiya et al., 2013a). It remains unknown how this phosphorylation controls BLUS1 function.

In the present study, we showed that the BLUS1 C-terminal region serves as a regulatory domain for kinase activity and that the phosphorylation of Ser-348 located within the regulatory domain is required for alleviating auto-inhibition. Furthermore, using constitutive signaling variants of BLUS1, we demonstrated that a decrease in intercellular CO_2_ concentration via red light-driven photosynthesis is required for stomatal opening. Moreover, a functional analysis of these BLUS1 variants revealed an unexpected negative regulation of stomatal opening via blue light-dependent dephosphorylation of H^+^-ATPase.

## Results

### BLUS1 C-terminal truncation affects stomatal responses to blue and red lights

**Figure 1:**
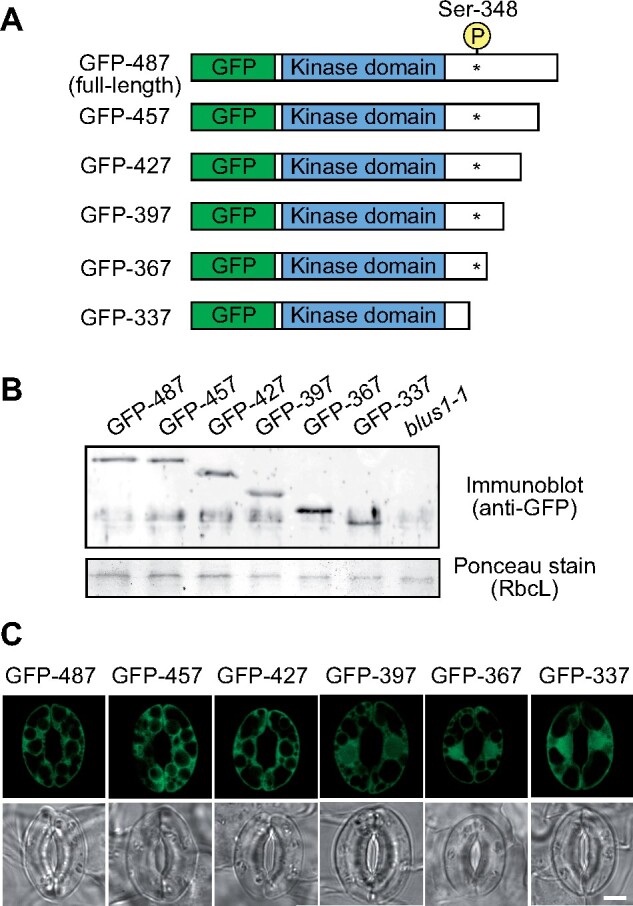
Expression of C-terminal truncated BLUS1 variants in the Arabidopsis *blus1-1* mutant. A, Schematic structure of the GFP-BLUS1 variants. Asterisks indicate the phosphorylation site in BLUS1 at Ser-348. B, Immunoblot analysis of the GFP-BLUS1 variants using anti-GFP antibodies. Each lane contained 5 μg of guard cell proteins. The Rubisco large subunit (RbcL) was used as the loading control. C, Subcellular localization of the GFP-BLUS1 variants in guard cells (upper panels) and the corresponding differential interference contrast images (lower panels). Bar = 5 μm.

To examine the functional role of the C-terminal region of BLUS1, we generated transgenic *Arabidopsis thaliana* plants expressing full-length BLUS1 (GFP-487) and a series of truncated BLUS1 proteins, each with deletions at the C-terminus, in increments of 30 amino acids (GFP-457, GFP-427, GFP-397, GFP-367, and GFP-337) (Figure 1A). Each BLUS1 variant fused with GFP was expressed in the *blus1-1* mutant under the control of the native promoter. Comparable expression of the GFP-BLUS1 variants in guard cells was verified by immunoblotting using anti-GFP antibodies (Figure 1B). GFP fluorescence of all GFP-BLUS1 variants was detected in the cytoplasm (Figure 1C). In addition, GFP fluorescence was detected in the nucleus in the GFP-367 and GFP-337 lines (Figure 1C), which can be attributed to the diffusion of small GFP fusion proteins through the nuclear pore complex (Wang and Brattain, 2007).

**Figure 2:**
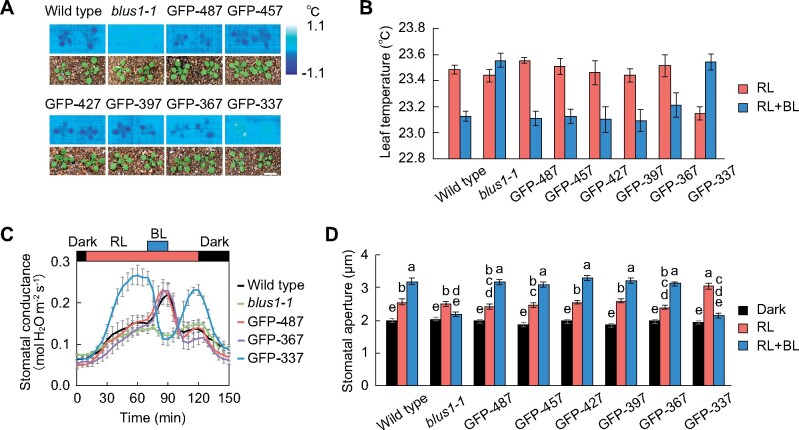
Effect of BLUS1 C-terminal truncation on light-dependent stomatal movement. A, Thermal image of blue light-dependent leaf temperature changes. Dark-adapted plants of wild-type, *blus1-1* mutant, and transgenic lines expressing C-terminal truncated BLUS1 were illuminated with red light (RL: 100 μmol m^−2^ s^−1^) for 50 min, and then blue light (BL: 10 μmol m^−2^ s^−1^) was superimposed on RL for 20 min. Subtractive images were generated by subtracting an initial thermal image taken before BL illumination from an image taken 20 min after BL illumination. Bar = 1 cm. B, Quantification of leaf temperature in the indicated lines. Data are presented as mean ± sem (*n* = 8). C, Light-dependent changes in stomatal conductance in intact leaves. The leaves of dark-adapted plants were illuminated with RL (300 μmol m^−2^ s^−1^) for 1 h, and then BL (10 μmol m^−2^ s^−1^) was superimposed as indicated. Data are presented as mean ± sem (*n* = 3). D, Light-dependent stomatal movement. Detached leaves were floated on stomatal opening buffer in the dark for 1 h. Thereafter, the leaves were illuminated with RL (300 μmol m^−2^ s^−1^) for 1 h, and then BL (10 μmol m^−2^ s^−1^) was superimposed for 20 min. Data are presented as mean ± sem (*n* = 75, pooled from triplicate experiments). Different letters indicate significant differences (ANOVA with Tukey’s test, *P* < 0.01).

We examined blue light-dependent stomatal opening by thermal imaging. Dark-adapted plants were exposed to a high fluence rate of red light to induce photosynthesis, and then to a low fluence rate of blue light to elicit blue light-dependent stomatal opening. In the wild-type plants, leaf temperature decreased after exposure to blue light by transpirational evaporation of water (Figure 2, A and B). Consistent with the findings of a previous study (Takemiya et al., 2013a), there was a slight increase in leaf temperature of the *blus1-1* mutant in response to blue light. C-Terminal deletion lines, GFP-457, GFP-427, GFP-397, and GFP-367, were as effective as full-length GFP-BLUS1 (GFP-487) in restoring stomatal opening. In contrast, the GFP-337-expressing transgenic line, which lacks the phototropin phosphorylation site, showed a lower leaf temperature under red light than other plants, and exhibited a large increase in response to blue light. No visible change was observed in the phenotype of these C-terminal deletion lines (Figure 2A).

We measured light-dependent stomatal opening in intact leaves using high-resolution gas-exchange techniques (Figure 2C). Exposure to strong red light increased stomatal conductance in the wild-type plants, and weak blue light superimposed on red light induced further stomatal opening; whereas the *blus1-1* mutant displayed slight stomatal closure in response to blue light. Transgenic plants expressing GFP-367 and GFP-487 in the *blus1-1* background fully recovered blue light-dependent stomatal opening, suggesting that the C-terminal region downstream of residue 367 is not required for blue light signaling. Unexpectedly, when irradiated with red light, the GFP-337 line exhibited a considerably higher stomatal conductance comparable to that of the wild-type plants under blue light. Conversely, blue light illumination caused significant stomatal closure in the GFP-337 line. These stomatal phenotypes were further confirmed by stomatal aperture measurements in the leaves in independent transgenic lines (Figure 2D; Supplemental Figure S1). Consistent with the findings of thermal imaging, stomatal opening in the GFP-457, GFP-427, and GFP-397 lines under red and blue light illumination was similar to that in the wild-type plants (Figure 2D; Supplemental Figure S1). Furthermore, we confirmed that stomatal density and size of the transgenic plants were similar to those of the wild-type and *blus1-1* mutant plants (Supplemental Figure S2).

### BLUS1 C-terminal region acts as an auto-inhibitory domain

**Figure 3:**
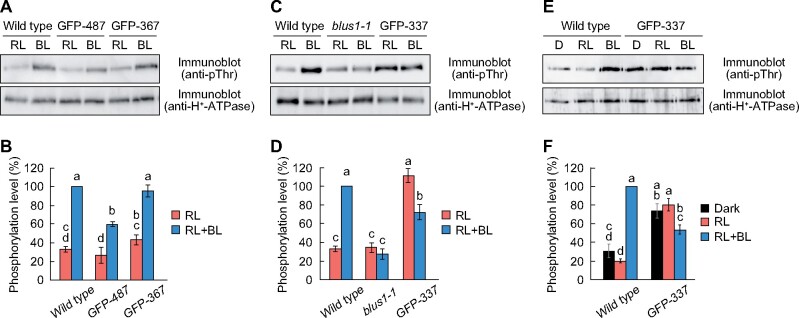
Effect of BLUS1 C-terminal truncation on blue light signaling. A–D, Phosphorylation of H^+^-ATPase in the BLUS1 C-terminal truncated lines. Guard cell protoplasts were illuminated with red light (RL: 300 μmol m^−2^ s^−1^) for 30 min, and then blue light (BL: 10 μmol m^−2^ s^−1^) was superimposed for 3.5 min. The phosphorylation and amount of H^+^-ATPase were detected by immunoblotting using anti-pThr947-AHA2 and anti-H^+^-ATPase antibodies, respectively. E and F, Phosphorylation of H^+^-ATPase in the GFP-337 line. Guard cell protoplasts were incubated in the dark for 30 min followed by illumination with RL (300 μmol m^−2^ s^−1^) for 30 min, and then BL (10 μmol m^−2^ s^−1^) was superimposed for 3.5 min. For (B), (D), and (F), the relative phosphorylation level of H^+^-ATPase was quantified using ImageJ software. Each value is expressed as a percentage of the phosphorylation level of wild-type plants under BL. Data are presented as mean ± sem (*n* = 3). Different letters indicate significant differences (ANOVA with Tukey’s test, *P* < 0.05).

H^+^-ATPase is activated via BLUS1-mediated blue light signaling, thus providing a driving force for stomatal opening (Kinoshita and Shimazaki, 1999; Takemiya et al., 2013a). As the GFP-337 line shows substantial stomatal opening under red light (Figure 2, C and D), we suspected that H^+^-ATPase might be activated by red light. To explore this possibility, we examined light-induced phosphorylation of H^+^-ATPase in guard cell protoplasts. H^+^-ATPase phosphorylation was apparent in the GFP-367 and GFP-487 lines in response to blue light (Figure 3, A and B). In contrast, the GFP-337 line showed increased H^+^-ATPase phosphorylation under red light, whereas superimposition of blue light elicited slight dephosphorylation (Figure 3, C and D). To verify that the increased H^+^-ATPase phosphorylation and stomatal opening in the GFP-337 line under red light were induced by the kinase activity of the GFP-337, we expressed a catalytic inactive form of GFP-337 (D157N) in the *blus1-1* mutant (Supplemental Figure S3, A and B) (Takemiya et al., 2016). We did not observe increased phosphorylation of H^+^-ATPase or stomatal opening in the GFP-337 (D157N) line (Supplemental Figure S3, C–F), suggesting that the kinase activity of GFP-337 is required for these responses. Unexpectedly, the GFP-337 line also showed increased H^+^-ATPase phosphorylation even in the dark (Figure 3, E and F), although it rarely opened the stomata under such conditions (Figure 2, C and D).

**Figure 4:**
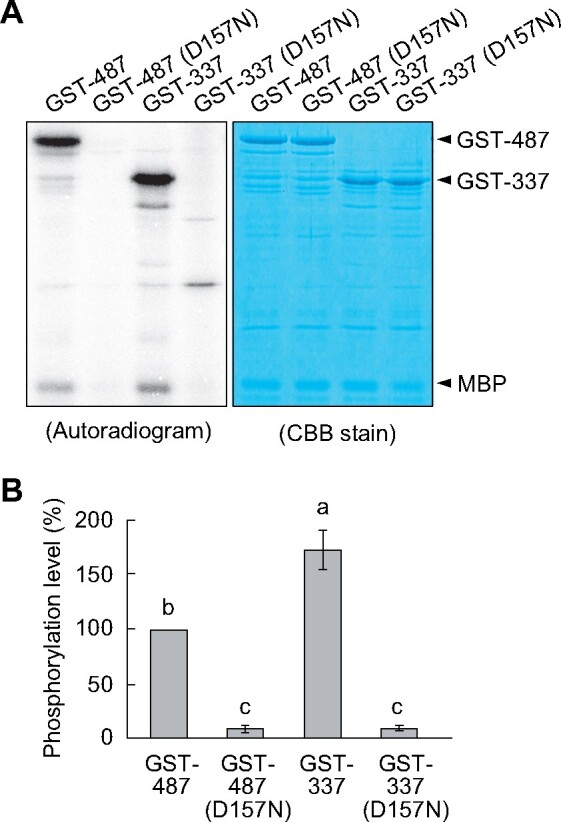
In vitro kinase assay of BLUS1 variants. A, Recombinant GST-BLUS1 and GST-337 were incubated with MBP in the presence of [γ-^32^P] ATP for 3 h. The phosphorylation of MBP and autophosphorylation of the GST-BLUS1 variants were visualized by autoradiography. B, Quantification of the relative phosphorylation level of MBP using ImageJ software. Data are presented as mean ± sem (*n* = 4). Different letters indicate significant differences (ANOVA with Tukey’s test, *P* < 0.01).

Given that the GFP-337 line showed H^+^-ATPase phosphorylation in the dark, we wanted to verify whether the deletion of the C-terminal region downstream of Glu-337 might result in the constitutive activation of BLUS1. For this purpose, we incubated recombinant GST-tagged BLUS1 variants with myelin basic protein (MBP) in the presence of [γ-^32^P] ATP. Both GST-487 and GST-337 showed autophosphorylation and MBP transphosphorylation activities (Figure 4A). Notably, GST-337 phosphorylated MBP more efficiently than GST-487 (Figure 4, A and B). In contrast, MBP phosphorylation was not observed in inactive GST-BLUS1 variants harboring the D157N mutation (Figure 4, A and B). Taken together, these data suggest that 30 amino acids, from Asp-338 to Glu-367, in the C-terminal of BLUS1 act as a regulatory domain that inhibits its kinase activity, and thereby regulate downstream signaling.

### Functional role of BLUS1 phosphorylation at Ser-348

**Figure 5:**
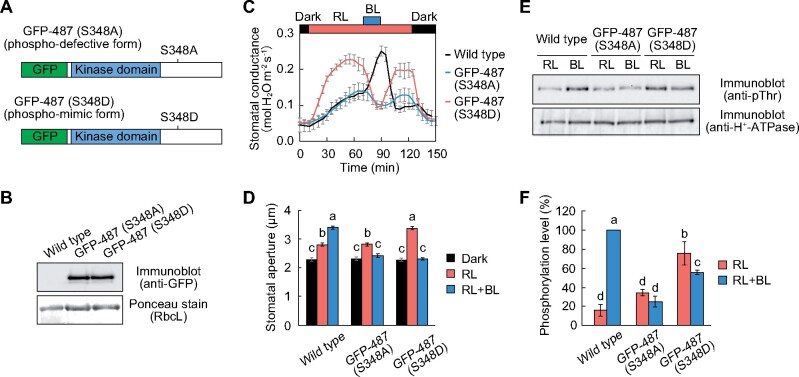
Effect of phospho-mimic mutation of BLUS1 on stomatal opening and blue light signaling. A, B, Schematic structure (A) and expression of GFP-487 (S348A) and GFP-487 (S348D) in the *blus1-1* background (B). Each lane contained 5 μg of guard cell proteins. RbcL was used as the loading control. C, Light-dependent changes in stomatal conductance. The leaves of dark-adapted plants were illuminated with red light (RL: 300 μmol m^−2^ s^−1^) for 1 h, and then blue light (BL: 10 μmol m^−2^ s^−1^) was superimposed. Data are presented as mean ± sem (*n* = 3). D, Light-dependent stomatal movement. Detached leaves were floated on stomatal opening buffer in the dark for 1 h. Thereafter, the leaves were illuminated with RL (300 μmol m^−2^ s^−1^) for 1 h, and then BL (10 μmol m^−2^ s^−1^) was superimposed for 20 min. Data are presented as mean ± sem (*n* = 75, pooled from triplicate experiments). Different letters indicate significant differences (ANOVA with Tukey’s test, *P* < 0.01). E, Phosphorylation of H^+^-ATPase in the GFP-487 (S348A) and GFP-487 (S348D) lines. Guard cell protoplasts were illuminated with RL (300 μmol m^−2^ s^−1^) for 30 min, and then BL (10 μmol m^−2^ s^−1^) was superimposed for 3.5 min. F, Quantification of H^+^-ATPase phosphorylation using ImageJ software. Each value is expressed as a percentage of the phosphorylation level of wild-type plants under BL. Data are presented as mean ± sem (*n* = 3). Different letters indicate significant differences (ANOVA with Tukey’s test, *P* < 0.05).

The phototropin-mediated phosphorylation site of BLUS1 at Ser-348 is located within the abovementioned regulatory domain (Takemiya et al., 2013a). Therefore, such phosphorylation could affect the function of the regulatory domain and thus blue light signaling. We generated transgenic plants expressing phospho-defective (S348A) and phospho-mimic (S348D) forms of GFP-BLUS1 in the *blus1-1* background (Figure 5, A and B). The expression of GFP-487 (S348A) did not enhance stomatal opening (Figure 5, C and D; Supplemental Figure S4A) and H^+^-ATPase phosphorylation (Figure 5, E and F) under blue light. In contrast, the GFP-487 (S348D) line exhibited enhanced stomatal opening in response to red light and closure in response to blue light (Figure 5, C and D; Supplemental Figure S4B). Furthermore, the GFP-487 (S348D) line showed increased phosphorylation of H^+^-ATPase under red light and slight dephosphorylation under blue light (Figure 5, E and F). Together, these results suggest that phosphorylation within the regulatory domain of BLUS1 may act as a switch to initiate downstream signaling and H^+^-ATPase activation.

### BLUS1-mediated stomatal opening requires a decrease in *Ci*

**Figure 6:**
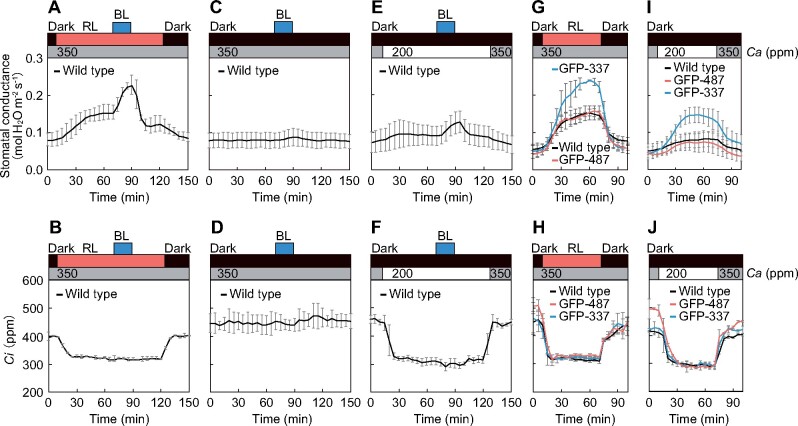
Light- and low CO_2_-induced stomatal responses. A–J, Changes in stomatal conductance (A), (C), (E), (G), and (I) and intercellular CO_2_ concentration (*Ci*) (B), (D), (F), (H), and (J) in the intact leaves of dark-adapted plants. Red light (RL: 300 μmol m^−2^ s^−1^) and blue light (BL: 10 μmol m^−2^ s^−1^) were illuminated as indicated. For (E), (F), (I), and (J), ambient CO_2_ concentration (*Ca*) was shifted from 350 to 200 ppm, and then returned to the initial level as indicated. For other measurements, the *Ca* was maintained at 350 ppm. Data are presented as mean ± sem (*n* = 3).

The expression of GFP-337 caused H^+^-ATPase phosphorylation under both dark- and red-light conditions (Figure 3, E and F); nevertheless, stomatal opening was not observed under dark conditions but was observed under strong red light (Figure 2, C and D). We hypothesized that this difference is caused by photosynthesis induced by red light (Shimazaki et al., 2007; Matthews et al., 2020). Similar to observations in many plant species (Doi et al., 2015; Matthews et al., 2020), stomata in Arabidopsis opened in response to weak blue light under strong red light, but scarcely opened without red light (Figure 6, A and C). The red light illumination decreased *Ci* to ∼300 ppm via mesophyll photosynthesis (Figure 6B), but weak blue light alone did not significantly reduce the *Ci* level (Figure 6D). Such a decrease in *Ci* could inhibit S-type anion channels, thus, leading to membrane hyperpolarization and stomatal opening (Roelfsema et al., 2002, Hiyama et al., 2017). Consequently, we examined stomatal conductance under low CO_2_ concentrations. Indeed, when *Ci* was decreased to levels similar to those under red light by manipulating the ambient CO_2_ concentration from 350 to 200 ppm in the dark, significant stomatal opening was induced by weak blue light in the wild-type plants (Figure 6, E and F). Consistent with the findings of previous studies (Karlsson, 1986; Assmann, 1988; Lascève et al., 1993), this blue light-dependent stomatal opening was enhanced under low CO_2_ concentration (Supplemental Figure S5, A and B).

The GFP-337 variant showed higher stomatal conductance than the wild-type plants and GFP-487 line under red light (Figure 6G), whereas both exhibited a similar *Ci* level under red light (Figure 6H). To determine whether such high stomatal conductance in the GFP-337 line could be attributed to both a decrease in *Ci* mediated by red light-induced photosynthesis and the high phosphorylation levels of H^+^-ATPase, we measured stomatal conductance under low CO_2_ conditions in the absence of red light. Interestingly, the GFP-337 line showed substantial stomatal opening when ambient CO_2_ was changed to 200 ppm, whereas the wild-type plants and GFP-487 line exhibited subtle changes in response to the CO_2_ shift (Figure 6, I and J). Thus, stomatal opening in the GFP-337 line appeared to be associated with the changes in *Ci*, in addition to the high phosphorylation levels of H^+^-ATPase. There was no significant difference in the CO_2_ assimilation rate under red light among the wild-type, GFP-487, and GFP-337 plants (Supplemental Figure S6A), and this can be attributed to the lack of differences in the *Ci* level (Figure 6H). Similarly, weak blue light illumination of the wild-type plants under strong red light enhanced stomatal opening (Figure 6A), but it did not alter *Ci* or CO_2_ assimilation rate (Figure 6B; Supplemental Figure S6B).

**Figure 7:**
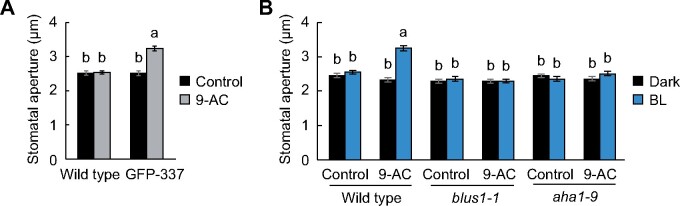
Effect of an anion channel blocker, 9-AC, on stomatal opening. A, Stomatal opening by 9-AC in the dark. Detached leaves were incubated in stomatal opening buffer with 50 µM 9-AC in the dark for 2 h. B, Stomatal opening by blue light and 9-AC. The leaves were incubated as described above, and then illuminated with blue light (BL: 10 µmol m^−2^ s^−1^) for 1 h. For (A) and (B), data are presented as mean ± sem (*n* = 75, pooled from triplicate experiments). Different letters indicate significant differences (ANOVA with Tukey’s test, *P* < 0.01).

To further address whether the stomatal opening under red light or low CO_2_ observed in the GFP-337 line was associated with the inhibition of anion channels, we examined the effect of an anion channel blocker anthracene-9-carboxylic acid (9-AC) on stomatal responses (Forestier et al., 1998; Hiyama et al., 2017). Application of 9-AC to leaves induced stomatal opening in the GFP-337 line even in the dark, whereas it did not affect stomatal responses in the wild-type plants (Figure 7A). Furthermore, when the leaves of the wild-type plants were pretreated with 9-AC, weak blue light elicited stomatal opening in the absence of red light (Figure 7B). In contrast, such blue light-induced stomatal opening in the presence of 9-AC was absent in the *blus1-1* and *aha1-9* (a knockout mutant of the major isoform of plasma membrane H^+^-ATPase in Arabidopsis guard cells) (Yamauchi et al., 2016) (Figure 7B). Taken together, these findings suggest that the simultaneous action of low *Ci*-regulated inhibition of anion channels and H^+^-ATPase activation is required for blue light-dependent stomatal opening.

### Arabidopsis* open stomata2* mutation causes a large stomatal opening irrespective of red light

**Figure 8:**
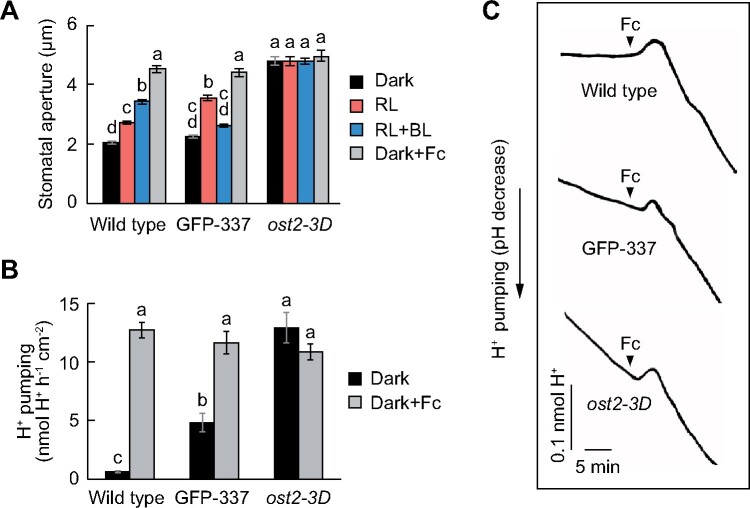
Comparison of stomatal opening and H^+^ pumping in the wild-type, GFP-337 transgenic, and *ost2* mutant plants. A, Light- and Fc-dependent stomatal movement. Detached leaves were floated on stomatal opening buffer in the dark for 1 h. Thereafter, the leaves were illuminated with red light (RL: 300 μmol m^−2^ s^−1^) for 1 h, and then blue light (BL: 10 μmol m^−2^ s^−1^) was superimposed for 20 min. For Fc-dependent stomatal opening, the leaves were incubated in stomatal opening buffer with 10 µM Fc in the dark for 1 h. Data are presented as mean ± sem (*n* = 75, pooled from triplicate experiments). Different letters indicate significant differences (ANOVA with Tukey’s test, *P* < 0.01). B, Maximum rate of H^+^ pumping. Data are presented as mean ± sem (*n* = 3). Different letters indicate significant differences (ANOVA with Tukey’s test, *P* < 0.05). C, Typical traces of H^+^ pumping in epidermal strips. Fc at 10 µM was added as indicated.

Previous studies have suggested that the Arabidopsis* open stomata2* (*ost2*) mutants, which express a highly active form of H^+^-ATPase AHA1, display a large and constitutive stomatal opening even in the dark. This finding is not consistent with the stomatal phenotype observed in the GFP-337 line (Merlot et al., 2007; Yamauchi et al., 2016). To explore this difference, we compared their stomatal responses to red and blue lights and fungal toxin fusicoccin (Fc), an activator of H^+^-ATPase. Consistent with the findings of previous studies (Merlot et al., 2007; Yamauchi et al., 2016), the *ost2-3D* mutant exhibited significant stomatal opening in the dark and under red and blue lights, and displayed a considerably larger stomatal aperture than the wild-type plants and GFP-337 line (Figure 8A). Furthermore, the application of Fc increased stomatal opening in both wild-type and GFP-337 plants, whereas the *ost2-3D* mutant showed comparable stomatal opening irrespective of Fc treatment (Figure 8A).

We further evaluated the H^+^-ATPase activity by measuring H^+^ pumping in the epidermal strips. Along with increased phosphorylation of H^+^-ATPase (Figure 3, E and F), we confirmed that the rate of H^+^ pumping was elevated in the GFP-337 line compared with that in the wild-type control in the dark (Figure 8, B and C). Furthermore, consistent with stomatal opening, the *ost2-3D* mutant showed a considerably higher rate of H^+^ pumping than the wild-type and GFP-337 plants in the dark and exhibited a comparable level of H^+^ pumping to that of epidermis treated with Fc (Figure 8, B and C). Together, these results suggest that both GFP-337 and *ost2-3D* mutant plants show increased H^+^-ATPase activity, but the *ost2-3D* plants presented considerably higher activity than that of GFP-337 line, and this might be responsible for the large stomatal opening in the *ost2-3D* mutant.

### Phototropins mediate H^+^-ATPase dephosphorylation and inhibit stomatal opening in response to blue light

**Figure 9:**
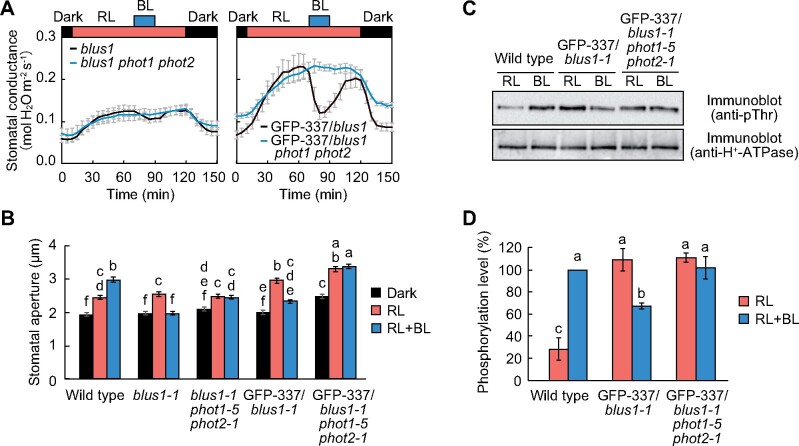
Phototropins mediate blue light-dependent stomatal closure and H^+^-ATPase dephosphorylation. A, Light-dependent changes in stomatal conductance. The leaves of dark-adapted plants were illuminated with red light (RL: 300 μmol m^−2^ s^−1^) for 1 h, and then blue light (BL: 10 μmol m^−2^ s^−1^) was superimposed. Data are presented as mean ± sem (*n* = 3). B, Light-dependent stomatal movement. Detached leaves were floated on stomatal opening buffer in the dark for 1 h. Thereafter, the leaves were illuminated with RL (300 μmol m^−2^ s^−1^) for 1 h, and then BL (10 μmol m^−2^ s^−1^) was superimposed for 20 min. Data are presented as mean ± sem (*n* = 75, pooled from triplicate experiments). Different letters indicate significant differences (ANOVA with Tukey’s test, *P* < 0.01). C, Phosphorylation of H^+^-ATPase in the transgenic lines. Guard cell protoplasts were illuminated with RL (300 μmol m^−2^ s^−1^) for 30 min, and then BL (10 μmol m^−2^ s^−1^) was superimposed for 3.5 min. D, Quantification of H^+^-ATPase phosphorylation using ImageJ software. Each value is expressed as a percentage of the phosphorylation level of wild-type plants under BL. Data are presented as mean ± sem (*n* = 3). Different letters indicate significant differences (ANOVA with Tukey’s test, *P* < 0.05).

The loss of function mutants of *BLUS1* did not exhibit stomatal opening but rather displayed stomatal closure in response to blue light (Figure 2) (Takemiya et al., 2013a). This indicates the presence of signaling mechanisms that downregulate stomatal opening in response to blue light, and suggests that the signaling pathway is independent of BLUS1. The transgenic line expressing GFP-337 in the *blus1-1* background showed a prominent stomatal closure in response to blue light (Figure 2, C and D). To ascertain whether the observed stomatal closure is mediated by phototropins, we generated a *blus1-1 phot1-5 phot2-1* triple mutant by crossing, and then analyzed light-induced stomatal movement. Blue light-dependent stomatal closure was not observed in the *blus1 phot1 phot2* triple mutant (Figure 9, A and B). Furthermore, when GFP-337 was expressed in the *blus1 phot1 phot2* triple mutant background, the plants exhibited enhanced stomatal opening under red light, but the blue light-dependent stomatal closure had disappeared (Figure 9, A and B). This indicates that phototropins mediate blue light-dependent stomatal closure.

Notably, the GFP-337 line showed partial dephosphorylation of H^+^-ATPase in response to blue light (Figure 3, C and E), and this might lead to the inactivation of H^+^-ATPase and thus suppress stomatal opening. Consequently, we examined the phosphorylation of H^+^-ATPase in guard cells of transgenic plants expressing GFP-337 in the *blus1 phot1 phot2* mutant. We found that H^+^-ATPase was substantially phosphorylated under red light, but blue light did not elicit the dephosphorylation of H^+^-ATPase in the triple mutant background (Figure 9, C and D). Thus, phototropins appear to mediate two opposite signaling pathways; one leading to H^+^-ATPase phosphorylation through BLUS1 and the other causing H^+^-ATPase dephosphorylation independent of BLUS1.

## Discussion

**Figure 10:**
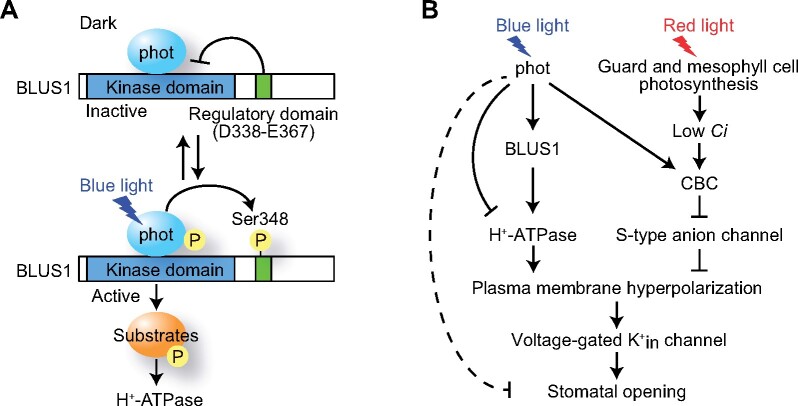
Model of blue light-dependent stomatal opening. A, Model of BLUS1-mediated blue light signaling. Without blue light, the C-terminal regulatory domain of BLUS1 represses its N-terminal kinase activity. Upon exposure to blue light, the light-activated phototropins autophosphorylate themselves and phosphorylate BLUS1 at Ser-348 within the auto-inhibitory domain. This leads to BLUS1 kinase activation, resulting in downstream signal transduction that activates H^+^-ATPase. B, Proposed model of coordinated control of stomatal opening by blue and red lights.

In the present study, we demonstrated that the C-terminal region of BLUS1 acts as a regulatory domain for its N-terminal kinase domain, and thereby regulates blue light signaling in stomatal guard cells. The functional analysis of Arabidopsis plants expressing C-terminal deletion mutants of BLUS1 in the *blus1* mutant background revealed that the truncation of C-terminal sequences after Glu-367 had no effect on blue light regulation of stomatal opening and H^+^-ATPase activation (Figure 2). Deletion of another 30 amino acid residues from Glu-367 resulted in the constitutive activation of H^+^-ATPase irrespective of blue light illumination (Figure 3, E and F). Furthermore, the in vitro kinase assay indicated that the deletion of these C-terminal sequences increased the phosphorylation activity of BLUS1 (Figure 4, A and B). These data suggest that the C-terminal 30 amino acid residues immediately downstream of the kinase domain of BLUS1 serve as a regulatory domain of auto-inhibition of the catalytic activity (Figure 10A).

In addition, we cannot exclude the possibility that the regulatory domain may regulate the interaction with downstream components. Recent research has shown that the Raf-like protein kinase BHP directly binds to BLUS1 and mediates blue light-induced activation of H^+^-ATPase (Hayashi et al., 2017). However, no obvious differences were detected in the binding of BHP to full-length and C-terminal truncated BLUS1 (Supplemental Figure S7A). Also, neither of these phosphorylated BHP (Supplemental Figure S7B). Thus, the BLUS1 regulatory domain is unlikely to modulate the binding or phosphorylation of BHP.

Our results indicate that phosphorylation at Ser-348 within the regulatory domain of BLUS1 by phototropins acts as a switch controlling the onset of blue light signaling and H^+^-ATPase activation (Figure 10A). Consistent with the previous study’s results (Takemiya et al., 2013a), the substitution of Ser-348 with Ala abrogated stomatal opening and H^+^-ATPase phosphorylation in response to blue light (Figure 5, C–F). In contrast, the present study’s results revealed that the phospho-mimic S348D mutation induced the phosphorylation of H^+^-ATPase without blue light (Figure 5, E and F). Importantly, the GFP-487 (S348D) line showed stomatal phenotypes similar to those of the GFP-337 line lacking a regulatory domain (Figures 2, 3, and 5). Mammalian Ste20-related proline alanine rich kinase (SPAK) and oxidative stress-responsive kinase 1 (OSR1) belong to the same subgroup of Ste20-related kinases as BLUS1, and the phosphorylation of the C-terminal domain by upstream with-no-lysine kinase (WNK) has been shown to enhance the N-terminal kinase activity (Filippi et al., 2011; Mehellou et al., 2018). Therefore, phosphorylation-based regulation of the kinase domain appears to be a common regulatory mechanism of the GCK-VI subgroup of Ste20-related kinases.

In the present study, the GFP-337 line showed enhanced stomatal opening under red light (Figure 2, C and D). Recent immunohistochemical analyses using Arabidopsis whole leaves indicated that plasma membrane H^+^-ATPase is phosphorylated not only by blue light but also upon exposure to red light in guard cells (Ando and Kinoshita, 2018). Therefore, we initially assumed that the GFP-337 line would exhibit higher phosphorylation of H^+^-ATPase than the wild-type plants under red light. Indeed, the GFP-337 line showed an increased H^+^-ATPase phosphorylation in guard cells under red light (Figure 3, C and D). However, increased phosphorylation of H^+^-ATPase was also observed in the dark in the GFP-337 line (Figure 3, E and F). Moreover, although the GFP-337 line showed a higher H^+^ pumping activity, it did not show stomatal opening in the dark (Figure 8). Irradiation of plants with strong red light reduces *Ci* via CO_2_ consumption by mesophyll photosynthesis (Hanstein et al., 2001), and this might inhibit the plasma membrane S-type anion channel encoded by SLAC1 (SLOW ANION CHANNEL-ASSOCIATED 1), through CO_2_ signal transduction (Karlsson, 1986; Assmann, 1988; Lascève et al., 1993; Roelfsema et al., 2002; Xue et al., 2011; Hiyama et al., 2017). Thus, such changes in *Ci* under red light would stimulate membrane hyperpolarization and stomatal opening. In support of this hypothesis, stomatal opening in the GFP-337 line was enhanced under dark conditions when *Ci* was decreased to levels under red light (Figure 6, I and J). Furthermore, 9-AC enhanced stomatal opening in the dark in the GFP-337 line (Figure 7A). Moreover, in the wild-type plants, weak blue light could induce stomatal opening without red light in the presence of 9-AC (Figure 7B). Taken together, our results indicate that blue light activation of H^+^-ATPase may not be sufficient to cause membrane hyperpolarization to trigger K^+^ influx and stomatal opening without deactivating the anion channels under physiological conditions. The findings also indicate that the *Ci* reduction induced by red light via mesophyll photosynthesis is a prerequisite for blue light-dependent stomatal opening. However, we note that 9-AC also has multiple potential targets, such as chloride channels and ATP-binding cassette transporters (Estévez et al., 2003; Ai et al., 2004), which play important roles in stomatal movements (Lee et al., 2008; Kang et al., 2010; Jossier et al., 2010; Wege et al., 2014). Considering the coordination of the ion transport between the plasma membrane and tonoplast (Horaruang et al., 2020; Cubero-Font and De Angeli, 2021), we cannot exclude the possibility that 9-AC interferes with anion transport at the tonoplast and promotes stomatal opening. Furthermore, electrophysiological analysis of the GFP-337 line will provide insight into the coordinated control of membrane transport and stomatal movements (Jezek and Blatt, 2017).

In contrast, several lines of evidence indicate that the *ost2* mutations cause increased H^+^-ATPase activity and stomatal opening even in the dark (Merlot et al., 2007; Yamauchi et al., 2016). These findings are contrary to the present results of GFP-337. The *ost2-3D* mutant showed considerably higher stomatal opening and H^+^ pumping than the GFP-337 line (Figure 8). These mutant phenotypes closely resemble the stomatal responses of plants treated with Fc (Figure 8). Thus, such increased H^+^-ATPase activity in the *ost2* mutant may result in more negative membrane potential than the threshold for the activation of inward K^+^ channels without inhibiting anion channels, as reported previously (Merlot et al., 2007).

Although the GFP-337 line exhibited stomatal opening in response to low *Ci* in the dark, the magnitude of opening was lower than that observed under red light (Figure 6, G and I). Similarly, a partial stomatal opening was observed when the leaves of the wild type were illuminated with weak blue light under low CO_2_ conditions without red light, but stomatal conductance was lower than that under blue light superimposed on red light (Figure 6, A and E). These differences can be attributed to the effect of red light-induced stomatal responses caused by mesophyll and guard cell photosynthesis, besides the *Ci*-driven response (Messinger et al., 2006; Lawson et al., 2008; Matrosova et al., 2015). Guard cell photosynthesis generates ATP for H^+^ pumping (Shimazaki et al., 1989; Tominaga et al., 2001; Suetsugu et al., 2014) or other metabolic processes involved in stomatal opening (Daloso et al., 2016; Santelia and Lawson, 2016). Furthermore, a diffusible signal originating from mesophyll photosynthesis has been implicated in stomatal opening (Mott et al., 2008; Fujita et al., 2013). Alternatively, malate formation, which acts as a counter-ion for K^+^ in guard cells, is also a possible candidate for enhanced stomatal opening. This is because malate formation is stimulated by weak blue light only in the presence of background strong red light (Ogawa et al., 1978).

Both BLUS1-null and kinase-dead mutants show a characteristic phenotype of blue light-dependent stomatal closure (Takemiya et al., 2013a). Such stomatal closure has also been observed in the double mutants, *blus1 phot1* and *blus1 phot2* (Takemiya et al., 2013a). In the triple mutant *blus1 phot1 phot2*, stomata did not close in response to blue light (Figure 9, A and B). These data suggest that phot1 and phot2 redundantly regulate stomatal closure via a signaling pathway separate from BLUS1.

Furthermore, our results show that lines expressing GFP-337 and GFP-487 (S348D) exhibit prominent stomatal closure in response to blue light (Figures 2, C and D, 5, C and D). Although it is unknown why these lines display such characteristics, they could be useful tools for further investigating this mechanism. Intriguingly, plasma membrane H^+^-ATPase was dephosphorylated in the GFP-337 line in response to blue light, whereas this dephosphorylation was not detected in the *blus1 phot1 phot2* triple mutant background (Figure 9, C and D). It is well established that H^+^-ATPase is activated via blue light-dependent phosphorylation (Shimazaki et al., 2007; Inoue and Kinoshita, 2017). In contrast, the present study revealed that blue light-activated phototropins also mediate the dephosphorylation of H^+^-ATPase, which might be involved in the attenuation and regulation of stomatal opening. Such blue light-induced dephosphorylation of H^+^-ATPase has also been observed in *Phaseolus vulgaris* pulvini and Arabidopsis hypocotyls (Inoue et al., 2005; Hohm et al., 2014).

Furthermore, although the GFP-337 and GFP-487 (S348D) lines displayed significant dephosphorylation of H^+^-ATPase in response to blue light, the phosphorylation level of H^+^-ATPase in these two lines under blue light was still higher than that observed in the wild-type plants under red light (Figures 3, E and F, 5, E and F). Nevertheless, the stomatal apertures in the GFP-337 and GFP-487 (S348D) lines under blue light were smaller than those in the wild-type plants under red light (Figures 2D, 5D). Thus, we do not exclude the possibility that phototropins also stimulate stomatal closure via other signaling pathways besides the pathway for H^+^-ATPase dephosphorylation. Further research is required to elucidate the unknown mechanisms of blue light-dependent stomatal closure and H^+^-ATPase dephosphorylation in guard cells.

Here, we summarize our findings and illustrate a model of blue and red light regulation of stomatal opening (Figure 10B). The blue light-activated phototropins induce the activation of H^+^-ATPase through BLUS1. Red light lowers the intercellular CO_2_ concentration via photosynthetic CO_2_ fixation, which inhibits S-type anion channels via CBCs. In addition, phototropins inhibit the anion channels via the CBC-mediated signaling pathway. Both H^+^-ATPase activation and anion channel inactivation may contribute to plasma membrane hyperpolarization, and this drives the uptake of K^+^ into guard cells via voltage-gated inward-rectifying K^+^ channels and thus promotes stomatal opening. Contrarily, phototropins mediate the dephosphorylation of H^+^-ATPase, which is independent of BLUS1. Furthermore, phototropins also appear to suppress stomatal opening via unknown mechanisms, other than the dephosphorylation of H^+^-ATPase. Further research is required to elucidate the coordinated control of stomatal opening by both blue and red lights, that is, BLUS1-mediated H^+^-ATPase activation, CBC-mediated SLAC1 inactivation, phototropin-mediated H^+^-ATPase dephosphorylation, and stomatal opening inhibition.

## Materials and methods

### Plant materials and growth conditions

*A. thaliana* wild-type (Col), *blus1-1* (Takemiya et al., 2013a), *phot1-5 phot2-1* (Kinoshita et al., 2001), *aha1-9* (SAIL_1285_D12; Yamauchi et al., 2016), *ost2-3D* (Yamauchi et al., 2016), and transgenic plants were grown on soil:vermiculite (1:1) for 4 weeks with 14 h light/10 h dark period under white light (50 µmol m^−2^ s^−1^). For thermal imaging, plants were grown on 0.8% (w/v) agar plates containing half-strength Murashige–Skoog salts (pH 5.7), 2.3 mM MES, and 1% (w/v) sucrose for 7 days with continuous white light illumination. The plants were then transferred to a soil:vermiculite (1:1) mixture and grown for 9 days with 13 h light/11 h dark under white light illumination.

### Construction of transgenic plants

The 1,474-bp promoter region of the *BLUS1* and *GFP* sequence was amplified from the pRI 101-AN *BLUS1pro:GFP-BLUS1* vector (Takemiya et al., 2013a) using the following primers: (forward) 5′-GGCCAGTGCCAAGCTTGCTTTAGGAATGTTGAAAGTATTCAGAG-3′ and (reverse) 5′-TACCCCCGGGGTCGACTCCACCTCCACCCTTGTACAGCTC-3′. The product was subcloned into the *Hin*d III/*Sal* I sites of the pRI 101-AN vector (TaKaRa) using the In-Fusion system (Clontech). The full-length and C-terminal truncated fragments of *BLUS1* were amplified using the following primers, where the first forward primer was used in all reactions: (forward) 5′-TGGAGGTGGAGTCGACATGGCTCGGAACAAGCTCGAG-3′ and (reverse) 5ʹ-TTCAGAATTCGGATCCTTAACCCAAAACACTATCTTTATC-3′ for 487, 5′-TTCAGAATTCGGATCCTTATTCTTCTCTGCTCTTCTCTTCCTTGTC-3′ for 457, 5ʹ-TTCAGAATTCGGATCCTTACACTTTCAGTTTCTCTAACACCAAATC-3′ for 427, 5ʹ-TTCAGAATTCGGATCCTTAGAGTTCATACCCCGTTATTGTGAC-3′ for 397, 5ʹ-TTCAGAATTCGGATCCTTATTCAGTAGCTGGGAACACTGGAC-3′ for 367, or 5ʹ-TTCAGAATTCGGATCCTTATTCTTCTTCTTCTTCTTCATCATCATCTCC-3′ for 337. The resulting products were inserted after the GFP sequence in the above vector using *Sal* I/*Bam* HI sites. Amino acid substitutions were performed using the QuikChange Site-Directed Mutagenesis Kit (Stratagene), and all amino acid substitutions were verified by sequencing. Each of the constructs was transformed into the *blus1-1* mutant with *Agrobacterium tumefaciens* strain GV3101.

### Measurement of stomatal opening

Stomatal conductance in intact leaves was measured using a gas-exchange system (LI-6400; Li-Cor) under the following settings: 350 ppm CO_2_, 24°C leaf temperature, 40%–50% relative humidity, and 200 µmol m^−1^ flow rate. To measure light responses, the leaves of dark-adapted plants were illuminated with red light (300 µmol m^−2^ s^−1^) for 1 h, and then blue light (10 µmol m^−2^ s^−1^) was superimposed on the red light background for 20 min unless otherwise indicated. To measure CO_2_ response, the ambient CO_2_ concentration was shifted from 350 to 200 ppm in the dark.

For stomatal opening measurement, detached leaves from dark-adapted plants were floated in stomatal opening buffer containing 5 mM MES-bistrispropane (pH 6.5), 50 mM KCl, and 0.1 mM CaCl_2_ for 1 h at 24°C in the dark, and then the stomatal aperture was measured (represented as “Dark”). The leaves were then illuminated with red light (300 µmol m^−2^ s^−1^) for 1 h (represented as “RL”), and blue light (10 µmol m^−2^ s^−1^) was superimposed for 20 min (represented as “RL+BL”). For Fc-induced stomatal opening, the leaves were treated with 10 µM Fc in the stomatal opening buffer for 1 h in the dark. For the 9-AC treatment, the leaves were incubated with 50 µM 9-AC in the same buffer for 2 h in the dark, and then illuminated with blue light (10 µmol m^−2^ s^−1^) for 1 h. After each treatment, the leaves were homogenized in a blender (Waring Commercial) and epidermal peels were collected using a nylon mesh. The stomatal aperture in the abaxial epidermis was measured using an inverted microscope (Eclipse TS100; Nikon).

Thermal imaging was carried out as reported previously (Takemiya et al., 2013a) with slight modifications. Dark-adapted plants were illuminated with red light (100 µmol m^−2^ s^−1^) for 50 min, and then blue light (10 µmol m^−2^ s^−1^) was superimposed. Leaf temperature was recorded using an infrared thermograph (H2640; NEC Avio Infrared Technologies). A subtraction image was obtained by subtracting an initial thermal image taken immediately before blue light illumination from an image taken 20 min after blue light using InfReC Analyzer NS9500 standard (NEC Avio Infrared Technologies).

### Guard cell protoplast isolation and immunoblotting

Guard cell protoplasts were isolated enzymatically from fully developed Arabidopsis leaves (Ueno et al., 2005; Takemiya et al., 2013b). To determine the phosphorylation level of H^+^-ATPase, guard cell protoplasts were incubated in 0.125 mM MES-NaOH (pH 6.0), 1 mM CaCl_2_, 0.4 M mannitol, and 10 mM KCl under red light (300 µmol m^−2^ s^−1^) for 30 min at 24°C, and then blue light (10 µmol m^−2^ s^−1^) was superimposed on the background red light, unless indicated otherwise. The reaction was terminated 3.5 min after the start of blue light illumination by adding trichloroacetic acid to the protoplast suspension. Immunoblotting was performed as reported previously (Kinoshita and Shimazaki, 1999; Takemiya et al., 2013b) with slight modifications. Antibodies against H^+^-ATPase (Kinoshita and Shimazaki, 1999) and phospho-Thr947 AHA2 (Hayashi et al., 2010) have been described previously. Antibodies against GFP were generated in rabbit using recombinant GST-GFP produced in *Escherichia coli*. The intensity of protein bands was quantified using ImageJ 1.48× software (National Institutes of Health).

### Measurement of H^+^ pumping

H^+^ pumping from epidermal strips was measured as described previously (Kinoshita et al., 2001) with some modifications. Abaxial epidermal strips (about 10 cm^2^) prepared from dark-adapted plants were incubated in 0.05 mM Mes-BTP (pH 7.0), 50 mM KCl, and 0.1 mM CaCl_2_ for 45 min in the dark at 24°C, and then 10 µM Fc was added. The medium pH was measured using a pH meter (SevenMulti; Mettler Toledo) equipped with a glass electrode (InLab Micro Pro; Mettler Toledo). The area of the epidermal strips was determined using ImageJ 1.48× software (National Institutes of Health).

### Confocal microscopy

The fluorescence images of GFP-BLUS1 variants in guard cells were collected using a confocal laser scanning microscope (Digital eclipse C1; Nikon). The wavelengths of excitation and emission were 488 and 515–530 nm, respectively.

### In vitro phosphorylation assay

Full-length and C-terminal truncated *BLUS1* were subcloned into the pGEX-2T vector (GE Healthcare), and the resulting constructs were introduced into *Escherichia coli* strain Rosetta 2(DE3) (Novagen). The recombinant GST fusion proteins were expressed and purified as described previously (Takemiya et al., 2013a), and used in the in vitro phosphorylation assay. The in vitro phosphorylation assay was carried out using a reaction mixture (30 µL) containing 20 mM HEPES-NaOH (pH 7.4), 5 mM MgCl_2_, 5 mM MnCl_2_, 3.3 µM ATP, 20 µCi [γ-^32^P] ATP (3,000 Ci mmol^−1^; PerkinElmer), 5 µg MBP, and 3 µg (37 pmol) GST–BLUS1 or 2.38 µg (37 pmol) GST-337 variant for 3 h at 15°C. The samples were then subjected to sodium dodecyl sulfate-polyacrylamide gel electrophoresis (SDS-PAGE) and stained with Coomassie Brilliant Blue. Protein phosphorylation was detected by autoradiography using Typhoon FLA 9500 (GE Healthcare).

### Statistical analysis

Data reported in this study are repeated at least three times and presented as mean ± standard error of the mean (SEM). The statistical analyses were performed using analysis of variance (ANOVA) followed by Tukey’s test in Excel 2007 (Microsoft) and Excel Toukei ver. 6.05 (Esumi). *P* value thresholds are shown as *P* < 0.05 or *P* < 0.01. Data for statistical analyses are shown in Supplemental Date Set S1.

### Accession numbers

Sequence data from this article can be found at NCBI and TAIR under accession numbers At4g14480 (*BLUS1*), At3g45780 (*PHOT1*), At5g58140 (*PHOT2*), At2g18960 (*AHA1*), and At4g18950 (*BHP*).

## Supplemental data

The following materials are available in the online version of this article.

**Supplemental Figure S1.** Light-dependent stomatal movements in independent transgenic plant expressing C-terminal truncated BLUS1.

**Supplemental Figure S2.** Stomatal size and density in transgenic plants expressing C-terminal truncated and amino acid-substituted BLUS1.

**Supplemental Figure S3.** Light-dependent stomatal responses in transgenic lines expressing kinase-dead form of GFP-337.

**Supplemental Figure S4.** Light-dependent stomatal movements in transgenic lines expressing phospho-defective and phospho-mimic variants of BLUS1.

**Supplemental Figure S5.** Effects of CO_2_ concentration on blue light-dependent stomatal opening.

**Supplemental Figure S6.** Net CO_2_ assimilation rate in Arabidopsis wild-type and BLUS1 C-terminal truncation lines.

**Supplemental Figure S7.** In vitro pull-down and kinase assays for the relation between BHP and C-terminal truncated BLUS1.

**Supplemental Data Set S1.** ANOVA table.

## Supplementary Material

koab067_Supplementary_Data

## Notes

S.H. and A.T. designed the research; S.H., S.Y., H.K., S.F., S.K., and A.T. performed the research; S.H., K.S., and A.T. analyzed the data and wrote the article.

The author responsible for distribution of materials integral to the findings presented in this article in accordance with the policy described in the Instructions for Authors (https://academic.oup.com/plcell) is: Atsushi Takemiya (take.pcs@yamaguchi-u.ac.jp).
