# Supplementary material for: A BLUS1 kinase signal and a decrease in intercellular CO2 concentration are necessary for stomatal opening in response to blue light
Source: Plant Cell. 2021 Mar 1;33(5):1813–27. doi: 10.1093/plcell/koab067 (PMC8254492; doi:10.1093/plcell/koab067)
Supplement: koab067_Supplementary_Data [file koab067_supplementary_data.zip › tpc.00720.2020-s01.pdf]

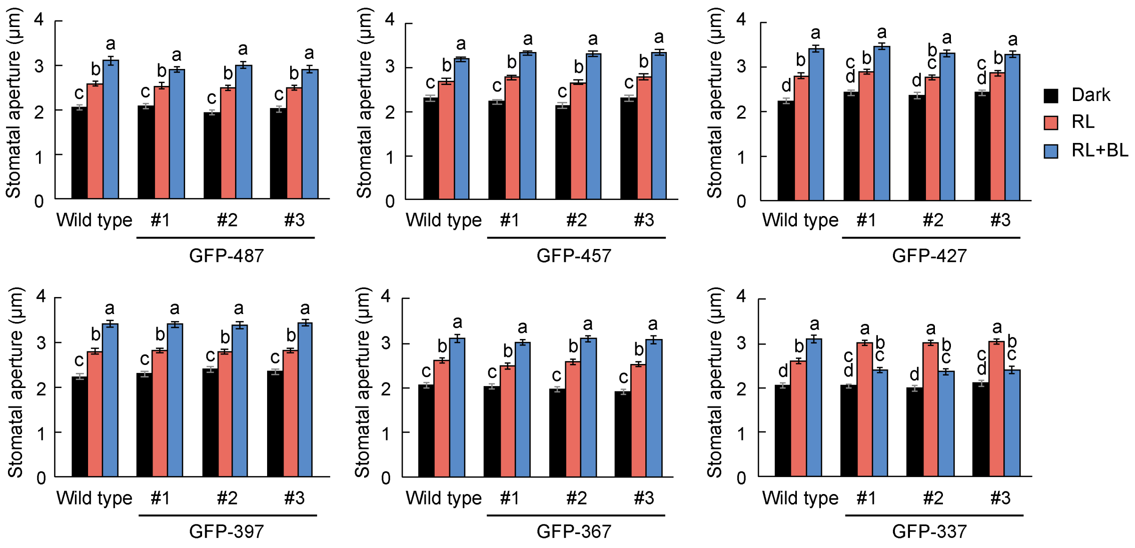

Supplemental Figure S1. Light-dependent stomatal movements in independent transgenic plant expressing C-terminal truncated BLUS1. Supports Figure 2.

Detached leaves were floated on stomatal opening buffer in the dark for 1 h. Thereafter, the leaves were illuminated with red light (RL: 300 μmol m^{-2} s^{-1}) for 1 h, and then blue light (BL: 10 μmol m^{-2} s^{-1}) was superimposed for 20 min. Data are presented as mean ± SEM (n = 75, pooled from triplicate experiments). Different letters indicate significant differences (ANOVA with Tukey's test, $P < 0.01$).

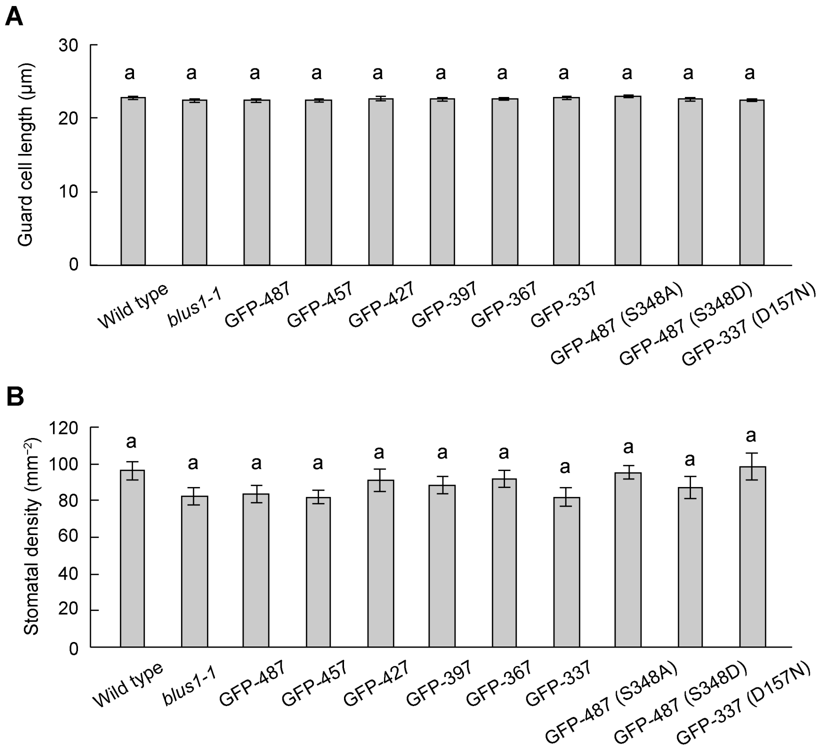

Supplemental Figure S2. Stomatal size and density in transgenic plants expressing C-terminal truncated and amino acid-substituted BLUS1. Supports Figure 2.

(A) and **(B)** Guard cell length **(A)** and stomatal density **(B)** in the abaxial epidermis of the wild type, *blus1-1* mutant, and transgenic lines. Data are presented as mean ± SEM (*n* = 60) **(A)** and (*n* = 32) **(B)**. The same letters indicate no significant difference (ANOVA with Tukey's test, P < 0.01).

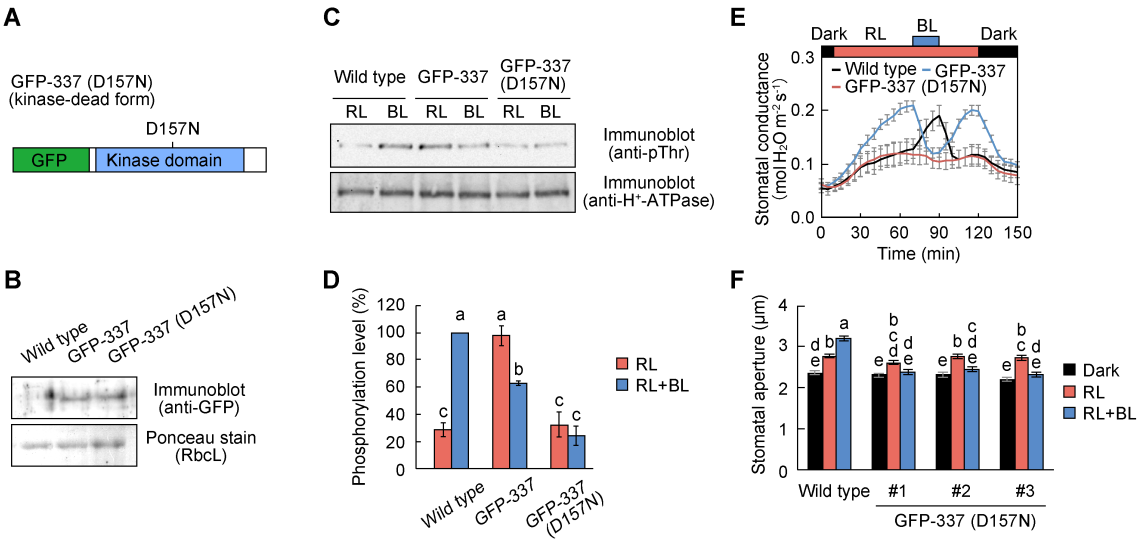

Supplemental Figure S3. Light-dependent stomatal responses in transgenic lines expressing kinase-dead form of GFP-337. Supports Figure 3.

(A) and **(B)** Schematic structure **(A)** and expression **(B)** of GFP-337 (D157N) in the *blus1-1* background. Each lane contains 5 μg of guard cell proteins. Rubisco large subunit (RbcL) was used as the loading control.

(C) and **(D)** Phosphorylation of H^+-ATPase. Guard cell protoplasts were illuminated with red light (RL: 300 μmol m^{-2} s^{-1}) for 30 min, and then blue light (BL: 10 μmol m^{-2} s^{-1}) was superimposed for 3.5 min. The phosphorylation and amount of H^+-ATPase were detected by immunoblotting using anti-pThr947-AHA2 and anti-H^+-ATPase antibodies, respectively. The relative phosphorylation level of H^+-ATPase was quantified using ImageJ software. Each value is expressed as a percentage of the phosphorylation level of wild-type plants under BL. Data are presented as mean ± SEM (n = 3). Different letters indicate significant differences (ANOVA with Tukey's test, $P < 0.05$).

(E) Light-dependent changes in stomatal conductance. The leaves of dark-adapted plants were illuminated with RL (300 μmol m^{-2} s^{-1}) for 1 h, and then BL (10 μmol m^{-2} s^{-1}) was superimposed as indicated. Data are presented as mean ± SEM (n = 3).

(F) Light-dependent stomatal movements in independent transgenic plants. Detached leaves were floated on stomatal opening buffer in the dark for 1 h. Thereafter, the leaves were illuminated with red light (RL: 300 μmol m^{-2} s^{-1}) for 1 h, and then blue light (BL: 10 μmol m^{-2} s^{-1}) was superimposed for 20 min. Data are presented as mean ± SEM (n = 75, pooled from triplicate experiments). Different letters indicate significant differences (ANOVA with Tukey's test, $P < 0.01$).

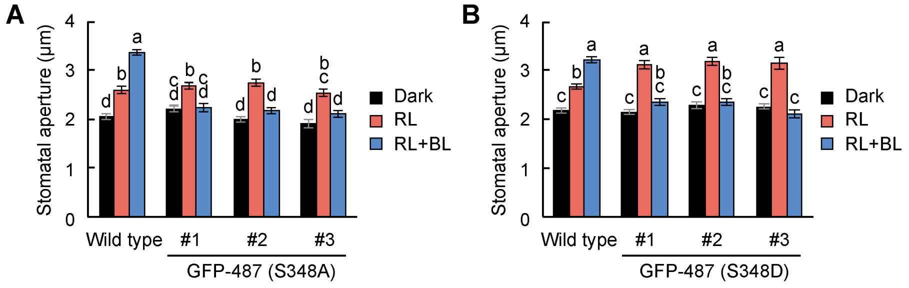

Supplemental Figure S4. Light-dependent stomatal movements in transgenic lines expressing phospho-defective and phospho-mimic variants of BLUS1. Supports Figure 5.

(A) and **(B)** Detached leaves from GFP-487 (S348A) **(A)** and GFP-487 (S348D) **(B)** were floated on the stomatal opening buffer in the dark for 1 h. Thereafter, the leaves were illuminated with red light (RL: 300 µmol m^{-2} s^{-1}) for 1 h, and then blue light (BL: 10 µmol m^{-2} s^{-1}) was superimposed for 20 min. Data are presented as mean ± SEM (n = 75, pooled from triplicate experiments). Different letters indicate significant differences (ANOVA with Tukey's test, $P < 0.01$).

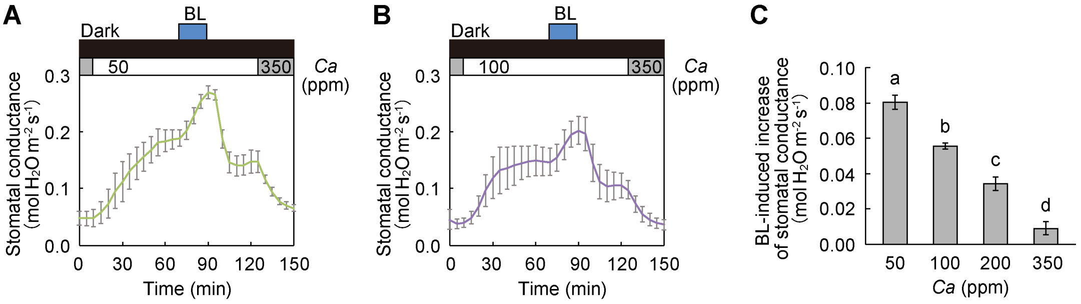

Supplemental Figure S5. Effects of CO_2 concentration on blue light-dependent stomatal opening. Supports Figure 6.

(A) and **(B)** Changes in stomatal conductance in response to blue light (BL: 10 μmol m^{-2} s^{-1}) in wild type. Ambient CO_2 concentration (*Ca*) was shifted from 350 to 50 ppm **(A)**, or 350 to 100 ppm **(B)**, as indicated. Data are presented as mean ± SEM (*n* = 3).

(C) Magnitude of blue light-dependent increase in stomatal conductance under different CO_2 concentrations. Data are presented as mean ± SEM (*n* = 3). Different letters indicate significant differences (ANOVA with Tukey's test, $P < 0.05$).

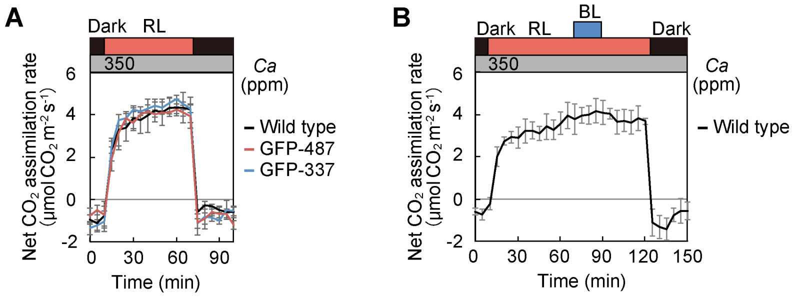

Supplemental Figure S6. Net CO_2 assimilation rate in Arabidopsis wild type and BLUS1 C-terminal truncation lines. Supports Figure 6.

(A) The leaves of dark-adapted plants were illuminated with red light (RL: 300 μmol m^{-2} s^{-1}) under 350 ppm CO_2 as described in Figures 6G and 6H. Data are presented as mean ± SEM (*n* = 3).

(B) The leaves of dark-adapted wild-type plants were illuminated with RL (300 μmol m^{-2} s^{-1}) and blue light (BL: 10 μmol m^{-2} s^{-1}) under 350 ppm CO_2 as described in Figures 6A and 6B. Data are presented as mean ± SEM (*n* = 3).

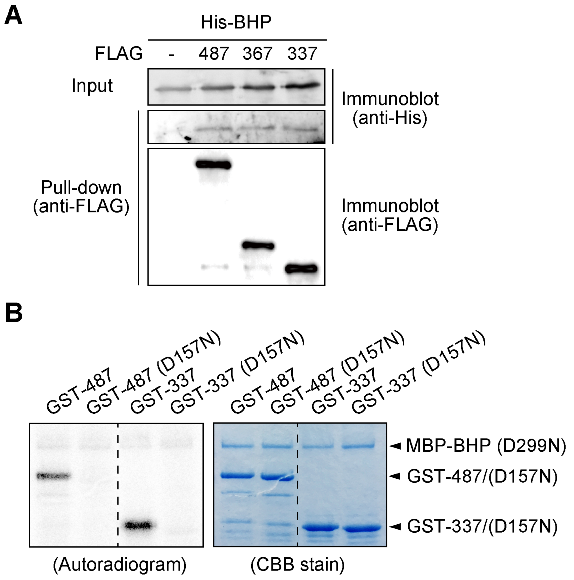

Supplemental Figure S7. In vitro pull-down and kinase assays for the relation between BHP and C-terminal truncated BLUS1. Supports Figure 4.

(A) In vitro pull-down assay. His-BHP and FLAG-tagged BLUS1 variants were synthesized by in vitro transcription and translation and incubated with anti-FLAG beads. The proteins bound to the beads were detected using anti-His and anti-FLAG antibodies.

(B) In vitro kinase assay. Recombinant maltose-binding protein (MBP)-tagged BHP and glutathione S-transferase (GST)-tagged BLUS1 variants were incubated with [γ-^{32}P] ATP for 3 h. Proteins were separated by SDS-PAGE, and phosphorylation was visualized by autoradiography.
